# Evolution of Biochip Technology: A Review from Lab-on-a-Chip to Organ-on-a-Chip

**DOI:** 10.3390/mi11060599

**Published:** 2020-06-18

**Authors:** Neda Azizipour, Rahi Avazpour, Derek H. Rosenzweig, Mohamad Sawan, Abdellah Ajji

**Affiliations:** 1 Institut de Génie Biomédical, Polytechnique Montréal, Montreal, QC H3C 3A7, Canada; neda.azizipour@polymtl.ca; 2 Department of Chemical Engineering, Polytechnique Montréal, Montreal, QC H3C 3A7, Canada; rahi.avazpour@polymtl.ca; 3 Department of Surgery, McGill University, Montreal, QC H3G 1A4, Canada; derek.rosenzweig@mcgill.ca; 4 Injury, Repair and Recovery Program, Research Institute of McGill University Health Centre, Montreal, QC H3H 2R9, Canada; 5 Polystim Neurotech Laboratory, Electrical Engineering Department, Polytechnique Montreal, QC H3T 1J4, Canada; 6 CenBRAIN Laboratory, School of Engineering, Westlake Institute for Advanced Study, Westlake University, Hangzhou 310024, China; 7 NSERC-Industry Chair, CREPEC, Chemical Engineering Department, Polytechnique Montreal, Montreal, QC H3C 3A7, Canada

**Keywords:** organ-on-a-chip, lab-on-a-chip, microfluidics, microengineering, BioMEMS, point-of-care, personalized medicine

## Abstract

Following the advancements in microfluidics and lab-on-a-chip (LOC) technologies, a novel biomedical application for microfluidic based devices has emerged in recent years and microengineered cell culture platforms have been created. These micro-devices, known as organ-on-a-chip (OOC) platforms mimic the in vivo like microenvironment of living organs and offer more physiologically relevant in vitro models of human organs. Consequently, the concept of OOC has gained great attention from researchers in the field worldwide to offer powerful tools for biomedical researches including disease modeling, drug development, etc. This review highlights the background of biochip development. Herein, we focus on applications of LOC devices as a versatile tool for POC applications. We also review current progress in OOC platforms towards body-on-a-chip, and we provide concluding remarks and future perspectives for OOC platforms for POC applications.

## 1. Introduction

Soon after the development of micro-electro-mechanical systems (MEMS), the potential of these miniaturized platforms for various applications in life science has been revealed. During the past few decades, interest in biological or biomedical MEMS (BioMEMS) has been drastically increased and it has found widespread applications in a various areas of biomedical and life science including diagnostics, therapeutics, drug delivery, biosensors and tissue engineering [1]. These integrated systems are also known as “lab-on-a-chip” (LOC) or “micro-total analysis systems” (μTAS). Microfluidic based LOC devices have often been notified as a landmark in biomedical research and life science [2,3]. However many microfluidic based devices which are currently categorized under BioMEMS do not have any electrical or mechanical components (e.g., DNA and protein arrays) [1].

In recent years, microfluidics technology with many advantages including precise control over the cellular microenvironment in very small volumes [4,5], merged seamlessly with cell biology and tissue engineering techniques [6,7]. This has enabled us to develop novel microengineered cell culture platforms [7,8].

OOC platforms which are microfluidic cell culture devices to mimic tissue- and organ-level physiology [9,10], have been developed very rapidly in the past few years. These platforms with great potential to advance our understanding about tissue and organ physiology [11], offer portable and cost-effective biomedical tools for diseases modeling [12,13], pharmaceutical research [14,15] and personalized medicine [5,6]. In OOC, the word “chip” roots from the original fabrication techniques [7,16] (e.g., a modified form of photolithography) which have been used in computer microchips manufacturing [17]. This allows us to control surface feature shapes and sizes on the nm to µm scale [18].

This paper focuses on development of LOC and OOC technologies from their origins. Here, we briefly describe LOC devices for POC application. We also highlight previous and recent progress in both areas of LOC and OOC and introduce some of the major pioneers in this market. Finally, by looking through specifications of LOC and OOC, we discuss future perspectives in the development of OOC platforms toward user friendly devices for drug discovery and POC applications.

## 2. BioMEMS

**Figure 1:**
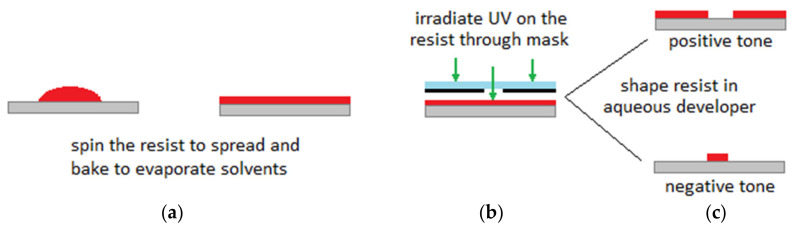
Process of photolithography: (**a**) Spinning a photoresist to spread and heat up to evaporate any solvents, (**b**) irradiating with UV light through a photomask, (**c**) positive/negative tone. Figure is reproduced from the Reference [3].

Dating back to the 1950s, new techniques in microfabrication technology developed rapidly when the planar technologies were introduced into the microelectronics [19,20]. At the beginning of the 1980s, with major progress in microelectronic systems and taking the advantages of miniaturization and parallel manufacturing, the concept of microelectromechanical systems (MEMS) appeared as the integration of mechanical and electrical functions into a single chip with small structures for various applications (e.g., biochemical applications and chemical engineering applications) [2,19]. Similar microfabrication techniques as those used in microelectronics to manufacture integrated circuits (IC) in the semiconductor industry, are used to fabricate MEMS microdevices [1]. Generally, by repeating particular orders of photolithography, etching techniques and thin-film deposition steps, fabrication of a micro/nano scale structure on planar substrates is being achieved. Figure 1 demonstrates the basics of the photolithography process. It starts with spinning a photoresist with a certain speed to spread to a desired plate thickness on the substrate. The next step is to heat up the photoresist to evaporate any solvents. The photoresist should be then irradiated with UV light while passed through a photomask. A post exposure bake may be needed to accelerate the curing of the photoresist. For the positive tone interaction, the areas which are exposed to UV radiation are removed after development. This is opposite for the negative tone. Interested readers are referred to References [21,22,23] for a review of microfabrication and microstructure formation technologies.

In the past few decades, various techniques to form micro scale structures have been developed. A brief description on the techniques which are more relevant with BioMEMS and microfluidics is provided in the following sections. During the 1980s, the “total chemical analysis system” concept emerged in analytical chemistry to propose the process of automation in analytical systems [2]. In the early 1990s, Manz et al. presented the concept of using planar fluidic devices to handle small volumes of liquid and established the field of “miniaturized total chemical analysis system” (µTAS) for this concept [24]. The high speed electrophoretic separation of fluorescent dyes [25,26] and amino acids which are fluorescently labelled [27] are the first examples of microchip analysis, which was developed in the early 1990s. As MEMS based devices have evolved, the interest in microsystems as new research tools for biomedical applications has significantly increased and different start-ups were founded to manufacture and take advantage of these microsystems in the life science field [19]. Due to their small size, capability to work on short time scale and ability to act under physiologically relevant conditions, MEMS devices provide the unique opportunity for fabrication of analytical platforms which are particularly attractive for biological applications [28].

BioMEMS is a subset of MEMS which has the biological or biomedical applications. These miniaturized devices use manufacturing techniques inspired from microfabrication technology. Processing, delivery, manipulation and analysis and/or construction of biological or chemical samples take place in these micro-devices [29]. Interest in BioMEMS applications such as diagnostics in DNA and protein micro-arrays, microfluidics platforms, pacemakers, biosensors, drug delivery systems etc. is increasing very rapidly [30,31]. Stimulating neural implants, retinal implants for therapy of blind patients and microneedles for vaccination to prevent suffering from physical pain are some examples of BioMEMS applications [1]. The progress of BioMEMS technology coupled with the recent advancements in biotechnology (e.g., genomics, proteomics, tissue engineering), provide exciting opportunities for advancing the applications of BioMEMS devices. BioMEMS for detection (e.g., antibody detection, bacterial detection, viral detection), analysis (e.g., identification of bacteria and antibiotic susceptibility), diagnostics (e.g., cancer and autoimmune diseases), monitoring (blood glucose monitoring in diabetics patients), drug delivery (e.g., administration of antibiotics), cell culture (e.g., OOC platforms) are some of the practical applications achieved by the advances in microtechnologies [1,29]. Just like the important role of microprocessors in the computer revolutions, BioMEMS devices have a significant role in the future of biomedical science. BioMEMS technology puts together the innovative talents of physicians, biological scientists, electrical, mechanical, chemical and materials engineers to develop miniaturized devices with various biomedical applications [6,16].

Three categories of materials can be used for fabrication of MEMS based devices for biological applications. The first group includes silicon, glass and other materials that originate in the electronic industry and have been used in the early MEMS devices [1]. The second group of materials is plastic and polymers (e.g., polydimethylsiloxane known as PDMS). Polymers due to their biocompatibility, low thermal and electrical conductivities, low cost, ease of fabrication, rapid prototyping and ease of surface modification are ideally suited for fabrication of BioMEMS [32,33,34]. We refer the interested readers to References [35,36,37] for polymer-based microfabrication techniques. The third group contains biological materials such as proteins, cells and tissues which can be used in BioMEMS devices [9,38]. However the use of biological materials is quite new. This category of materials offers many attractive opportunities in biomedical area (e.g., tissue engineering, OOC) [6,17].

BioMEMS and μTAS are subsets of MEMS devices. Even though BioMEMS are more focused on micro-fabricated devices for biological applications, they have significant overlap with μTAS which are basically more dedicated to the integration of the sequence of laboratory steps to accomplish chemical analysis. Such a small platform may gather the whole laboratory functions into a chip format. The idea of LOC has been developed after realizing that applications of μTAS technologies are not just limited to analytical purposes. Accordingly, LOC devices also are a subset of MEMS. LOC platforms use microfluidics, which is the science of manipulation of extremely small volumes of liquids. LOCs are integrated microfluidics platforms to perform multiple laboratory processes into a single chip. In the following section, we will review microfluidic platforms and their applications in the biomedical field.

## 3. Microfluidics

**Figure 2:**
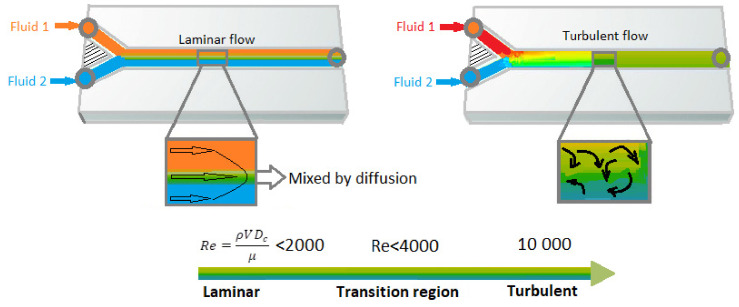
Laminar versus turbulent flow in the microfluidic channels. Figure is reproduced from Reference [44].

The annual number of new publications on the topic of microfluidics is increasing rapidly and continuously every year [39]. The investigation of fluid transport in plants is a good bio-mimicking example for studying of fluid mechanics in micro-channels, [40] on which it focuses on various specific characteristics of the dynamics of viscous flows in very small capillary tubes. However, because of the advances in microfabrication methods, the subject has received immense attention in recent decades [3,41]. A microfluidic platform consists of micro-scale fluid handling compartments such as channels, valves, reservoirs, membranes etc. which enable integrated, automated, parallelized and miniaturized biochemical analysis in a consistent and easy manner. Microfluidics with its specific characteristics such as small size and laminar flow pattern offer new abilities in terms of spatiotemporal control of molecules [19,42,43], enabling biomedical devices to decrease the size and increase precise control over the platform. Laminar flow regime happens at very low fluid speed or low Reynolds-number (“Re”). The Re number is a critical dimensionless number in fluidic dynamics which is used to characterize the behavior of the fluid in the system [2]. The Re number is characterized by the ratio of inertial forces to viscous forces [4]. Since viscous forces tend to keep fluid steams moving very smoothly over each other without chaotic mixing, when the viscous forces are dominant (at low Re), a series of parallel fluid streams appears without mixing between them. This type of fluid flow is known as laminar flow. However, at high Re, inertial forces are dominant which results in unexpected movements and chaotic mixing between the fluid streams. This type of fluid flow is known as a turbulent flow [3,44]. Figure 2 demonstrates the schema of the laminar and turbulent flow. Fluids behave in a different way at the microscale than they do at the macroscale. One of the most important differences between macroscopic and microfluidic systems is the type of flow in microfluidic systems [45]. Generally, the flow is turbulent at the macroscale (Re > 1000), while at the microscale laminar flow is dominant. In microfluidics, fluids flow in parallel patterns without any radial, axial and tangential mixing due to the absence of turbulent vortexes and mass transfer can only happens through the interface between the molecules of the fluid layers by diffusion. This type of flow, as mentioned, is known as laminar when the viscous forces are dominant on inertia forces which are characterized by smooth flow (Re < 1) as oppose to the characteristics of turbulent flow. Thus, microfluidics provide precise and spatiotemporal control over the fluid by providing a laminar regime over the system [4].

Surface tension is another important feature in microfluidic systems. The small amount of fluids in microfluidic systems and big surface to volume ratio will result in insignificant gravitational and inertial forces and significant surface interactions such as capillary and hydration forces in these systems. Therefore, surface tension becomes strongest and dominant in such systems [45].

Culture bottles which have been in use since almost 1850 [39] and culture dishes which were introduces by Petri in 1887 [46] are some of the basic classical liquid handling tools for biological analysis and diagnostic assays. These traditional liquid handling tools have the potential of high–throughput sample processing and are easy to handle [6,39], but lack of portability, lack of automation and high expenses to have a complex automated laboratory are some of their disadvantages [39]. Microfluidic devices have advantages to overcome these limits such as simpler automation, use of less sample volume, portability, simultaneous and parallel tests, faster analysis, lower cost per assay etc. [19,44] and compete well against these conventional tools.

Microfabricated micro-channels have been used by many researchers in this filed to accomplish capillary electrophoresis assays [47], to observe deformation of red blood cell membrane under hydrodynamic flow [48], in chemical micro-reactors [49], to transport living cells [50] and micromixers [51], among other applications.

Microfluidics have been raised from different origins of microelectronics, molecular biology, and chemical analysis [2,52,53]. One of the origins of microfluidics is microelectronics where microfabrication techniques such as photolithography have been shown to be very successful in microelectronics and MEMS devices. Microfluidics have been developed as a branch of MEMS devices which are specialized in liquid handling. The initial work in microfluidics has used silicon and glass as materials and microelectronic manufacturing techniques for the fabrication of micro-devices. However these materials are favorable when chemical or thermal stability is required, but neither silicon nor glass has the appropriate requirements such as gas permeability, full compatibility, and desired wettability for biological assays.

The other contribution to develop microfluidics systems has been raised from molecular biology. Genomics, Proteomics and other areas related to molecular biology (e.g., high-throughput DNA sequencing) require more sensitive and high throughput analytical methods. The ancient route of microfluidics comes from chemical and biochemical analysis. Chemical analysis techniques such as gas-phase chromatography (GPC), high-pressure liquid chromatography (HPLC) and capillary electrophoresis (CE) merged with the power of laser in optical detection to achieve sample analysis in low volume with high sensitivity. However, the number of commercialized microfluidics platforms in practical applications still is quite low and they have not been widely used yet, but microfluidics is very well established in academia for the development of new methods and various applications in biomedical areas [6,9].

At the beginning of the 1950s Elmqvist patented the first practical Reyleigh break-up ink-jet platform by efforts to distribute very small volume of liquids (nanoliter and picoliter) [54]. This innovation provided the basis for ink-jet techniques which are already used in printers. Later, in the year 1979, Terry et al. developed a miniaturized gas analysis platform based on the principles of gas chromatography (GC), using photolithography and chemical etching techniques on a silicon (Si) wafer [55]. Manz et al. by using Si-Pyrex technology have developed the first high-pressure liquid chromatography column device. At the beginning of the 1990s, several miniaturized microfluidics structures [56,57] have been developed by the techniques used in the fabrication of micro-scale structures in silicon [39]. Later, simple and easy to operate microfluidic devices based on capillary liquid transport have been developed. Test strips for drug abuse [58], pregnancy tests [59], cardiac markers [60] are among the first few commercial microfluidic devices which obtained a significant market and still have high sales potential.

Following the efforts towards miniaturization to reduce the time of analysis and to have better performance, the newly emerging concept of “micro total analysis systems” (µTAS) which later expanded to “LOC” appeared [24]. In the following section, we discuss about microfluidics application to develop LOC devices.

## 4. Lab-on-a-Chip

Lab-on-a-Chip (LOC) based-devices integrate multiple laboratory functions on a single chip of only a few square millimeters to a few square centimeters in size. These platforms provide miniaturized, automated, integrated and parallelized chemical and/or biological analyses [61] which can offer cheaper, faster, controllable and higher performance of bio-chemical assays at a very small scale when compared with conventional laboratory tests. These microengineered devices are capable of handling extremely small fluid volumes down to less than a few picoliters. [3]. Just a few micro-droplets of whole blood, plasma, saliva, tear, urine or sweat have the potential to be tested in these miniaturized platforms for medical diagnostics [62]. This last is highly important in many clinical trials and biomedical research where usually very small volumes of patient samples are available. In another side, automation with eliminating the human interfering parameters can increase the confidence in the analysis [63].

A clear example that demonstrates the principle of LOC is the portable blood test device which has been developed by the Biosite Company. Just a drop of patient’s blood is required to displace into the reservoir of the device then the entire of the assay takes place on a single platform and the diagnostic can be done in only a few minutes [19].

Other biomedical applications for LOC have been reported including proteins and DNA detection [18], hormone detection [64], pathogen detection [65]. In the following section, we focus on the application of LOC platforms in POC diagnostic devices.

### Lab-on-a-Chip Devices for Point-of-Care Diagnostics

Point-of-care (POC) diagnostic platforms are small medical devices which provide diagnostic results quickly in the easiest way [41]. However, these diagnostic procedures can be performed by healthcare professionals, but working with these devices does not need trained specialists and the tests can be done by the patient in a range of settings including home, laboratory, hospital or clinic. Increasingly, the need for the fast diagnostic of acute diseases such as acute myocardial infarction and for home care testing such as blood glucose monitoring in diabetic patients, has grown the interest to develop POC systems. The high surface area to volume ratio in microfluidic systems results in a significant decrease in the time of analysis in LOC for POC testing [66]. This provides the chance for rapid diagnosis and receipt of treatment as soon as possible at the point of care. Moreover, non-expert users can easily work and obtain the test results with these POC devices.

Lateral flow tests or capillary driven test strips which have been well known since the 1960s [61], are the major class of POC systems which use a membrane or paper strip to confirm the presence or absence of a target analyte such as host antibodies or pathogen-antigens. By adding a small volume of sample, capillary action [39] will be induced and the sample moves along the channel passing through the membrane where immobilized antibodies and labels have been stored. If the targeted particles are present in the sample, it will bind to the immobilized antibodies and labels and continue to move along the device. As the sample moves, the binding reagents which are located on the membrane will attach to the targeted compound at the test line. The test results can be read out qualitatively where a colored line forms, or quantitatively where the device has been combined with reader technology to provide results [66,67].

These automated and on-site diagnostic devices provide cost-effective, easy to handle and disposable tools to detect different biomarkers including proteins, nucleic acids, cells and metabolites such as glucose, urea nitrogen, lactate, etc. [66]. Test strips for detection of infectious diseases such as pneumonia [68] and influenza [66], also for detection of sexually transmitted infections like syphilis and HIV [41], are some of the examples of lateral flow tests in POC. In developing countries, due to the little or no medical infrastructure, the mortality rate from infectious disease is quite high [69,70]. Therefore, POC systems by fast detection of infectious diseases significantly help to increase patient survival rate [18].

**Figure 3:**
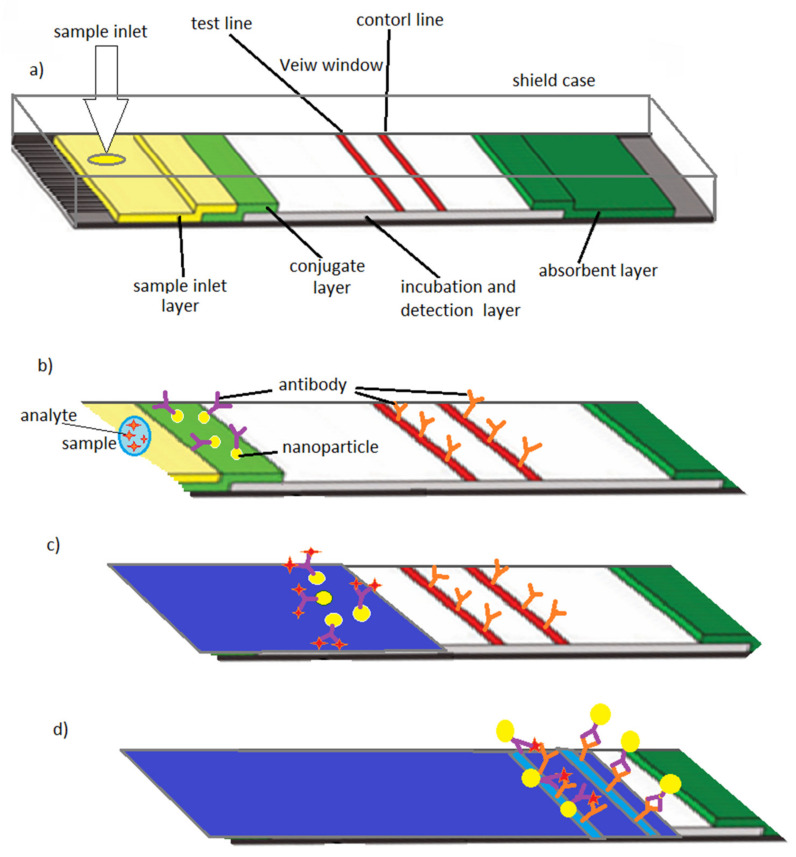
Lateral flow assay schematic design: (**a**) A lateral flow test strip including sample inlet, sample layer, conjugate layer (i.e., reactive agents and detection molecules), incubation, detection zone and final absorbent layers including test and control lines (i.e., analyte detection and functionality test), (**b**) introduction of sample into the test strip via sample inlet, (**c**) antibodies conjugated to labeled nanoparticles start to bind to the analyte, (**d**) antibodies with antigens bind to the test line and antibodies without antigens bind to the control line. Reproduced from Reference [39].

The test strips for glucose-level monitoring [71] for glycemic control and the lateral-flow immunoassay [72] for home pregnancy test are some of the well-established early examples of POC systems which obtained a remarkable market. Based on the first capillary driven immunoassay system that was introduced in the later 1970s [39], the “over-the-counter pregnancy test” was developed and commercialized in the 1980s [73]. The device consists of a sample inlet, a sample layer, a conjugate layer, incubation and detection layer, a detection window and an absorbent layer. When the patient’s sample (urine) is introduced into the sample layer through the device’s inlet, it moves by capillary forces. In the conjugate layer, where the antibodies have been stored, binding between antibodies and sample’s antigens (human chorionic gonadotropin or hCG) takes place. As the sample is transported into the detection layer, this binding reaction continues. Another type of antibody on the test line catches the particles which are coated with antigens. There is a third type of antibody on the control line to catch particles which did not bind to antigens. The presence or absence of hCG (pregnancy hormone) in the sample will be detected by the detection line, while the control line, which appears on every test, demonstrates that the test works properly. Within minutes, the result appears in detection window indicating whether or not hCG hormone has been detected in the sample [39,74] (Figure 3).

**Figure 4:**
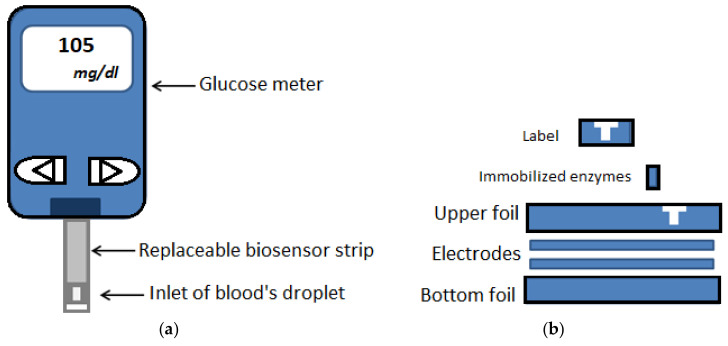
Blood glucose monitoring system: (**a**) Scheme of a commercial blood glucose test device, (**b**) different layers of a biosensor test strip. Reproduced from Reference [76].

Ionic blood chemicals and metabolites can be used as biomarkers to determine various health conditions such as liver disease, diabetes etc. Glucose monitoring systems for management of diabetes, have occupied the majority of the biosensor market [75]. Blood glucose monitoring devices (Figure 4) that significantly improve diabetic patients’ lives, also perform on membranes but its analytical method is different from lateral flow immunoassay as it use signal amplification by a redox enzyme. Reaction between glucose oxidase in the test strip and the glucose in the blood will produce an electrical current which determines the concentration of the glucose in the sample and provides numerical results for readout by the meter [41].

POC diagnostic devices have significant impacts in public health of developing countries. Although, low power consumption, cost-effective analysis, fully-automated test, ability to use unprocessed specimen (e.g., whole blood), being highly user-friendly, provide interpreted results quickly and less challenges regarding to transportation and storage are some of the parameters which should be given more attention to design of POC devices for resource-limited settings in the developing world. Table 1 provides a list of current microfluidics companies which provide commercialized LOC-based point of care diagnostic devices.

Increasing the interests towards microfluidics and its applications in biomedical areas in the last two decades led to the emergence of novel fabrication technology using biocompatible polymers for biological applications [6,16]. The incorporation of microfluidics with MEMS manufacturing techniques has provided the possibility of producing delicate molds for casting microfluidic patterns by use of a biocompatible polymer such as PDMS. PDMS has unique properties including gas permeability, biocompatibility, optical transparency, elasticity, low cost and ease of microfabrication with soft lithography technique [6,9]. These advancements in microengineering have led to innovate new generation of cell culture platforms known as OOC. In the following section, we highlight current achievements in this field.

## 5. Organ-on-a-Chip

As mentioned above, microfluidic cell culture platforms by providing a dynamic physiological microenvironment for cells and precise control over small amount of sample, offer unique advantages over traditional cell culture techniques (e.g., culture in Petri dishes) and static 3D cell culture models (e.g., scaffold techniques and scaffold free techniques). The majority of research relies on monoculture cell signaling and cell-cell interactions in two dimensions. However, cellular interactions in three dimensions at the tissue and organ levels have significant impacts in cellular responses to environmental cues [7,77]. Conventional 2D cell-cultures are not able to mimic the 3D structure, mechanical properties and biochemical microenvironment that cells experience in a living organ [7,78]. Various static 3D cell-culture models (e.g., bioreactors, spheroid and gel based cell culture techniques) were developed over 50 years ago to overcome the limitations related to 2D cell-culture [6]. Compared with conventional 2D models, 3D cell culture models better simulate the 3D microenvironment that cells normally experience in vivo and advanced our understanding of the cell behavior in better bio-mimicking tissue models [12]. On the other hand, microfluidic cell culture platforms provide better mimicking of a more sophisticated manner of cell culture and analysis at the microscale [79]. As compared to static cell culture models, microfluidic cell culture platforms are able to emulate the dynamic microenvironment for the cells when macroscopic cell culture models fail to reproduce the microenvironment of the cells as it is in vivo. Microfluidic cell culture based platforms offer the possibility of creating biochemical gradients of metabolites, soluble factors, cytokines and other molecules in the cellular microenvironment [6]. The specific characteristics of microfluidic systems, such as the laminar flow pattern, can provide sustained biochemical gradients. For instant, Han et al. developed an in-vivo like inflammatory model in a microfluidic platform to characterize neutrophil migration behavior influenced by a gradient of two chemoattractants. This study showed that neutrophils demonstrate different responses to the chemoattractants [80]. With other unique benefits such as decrease in reagent consumption, smaller volume of samples, decrease the risk of contamination, and reproducibility, microfluidic based culture models offer unique in vitro platforms for high throughput cell culture assays [81]. Table 2 illustrates an overview of the significant advantages of microfluidic cell culture versus macroscopic cell culture models (e.g., 2D cell culture and static 3D cell culture models).

“OOC” platforms are new generation of 3D cell culture models that better emulate the dynamic, physicochemical, biochemical and microarchitecture properties of the microenvironment of living organs. These microfluidic cell culture platforms recapitulate the cellular microenvironment, tissue–tissue and cell–cell interface interactions, spatiotemporal, biochemical gradients and biomechanical properties of a whole living organ with the aim of mimicking the smallest functional unit of an organ. 

**Figure 5:**
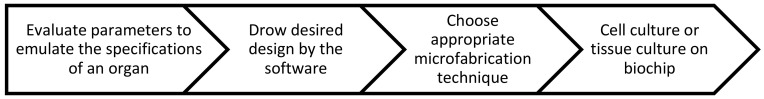
General process to fabricate a microfluidic OOC platform. Design, microfabrication, tissue culture and biological assays are the main steps to develop an OOC microfluidic platform for biological or pharmaceutical tests.

Figure 5 represents the general process to fabricate a microfluidic OOC platform. The principles to manufacture OOC based platforms are almost similar. Basically, after considering various parameters to emulate the specifications of a specific organ, the desired design would be drawn with a design and drafting software (e.g., AutoCAD, CATIA). Later, an appropriate microfabrication technique (e.g., photolithography, stereolithography, soft lithography etc.) according to the aims of the device will be used to fabricate the device. Cell culture or tissue culture will be performed on the biochip in order to mimic the functionality of a specific organ and to perform biochemical or biophysical assays and drug testing.

In the following section, we highlight the current advancements in OOC platforms.

### 5.1. Current Organ-on-a-Chip Platforms

#### 5.1.1. Lung

**Figure 6:**
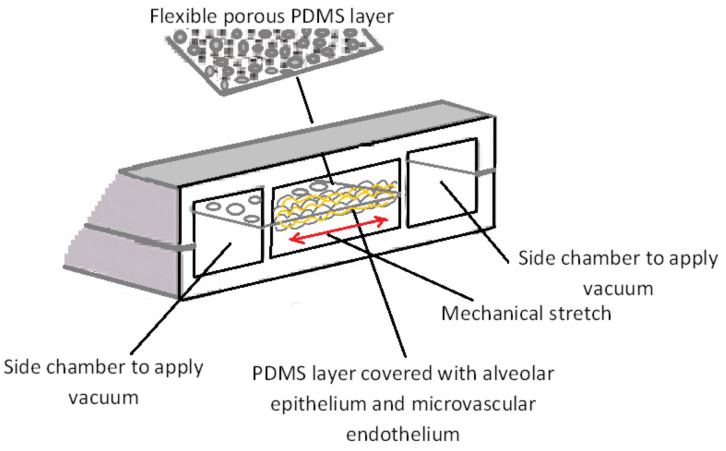
Schematic design of the microengineered lung-on-chip model developed by Huh et al. [12]. An alveolar-capillary interface constructed on a flexible and porous PDMS membrane. By applying vacuum to the side chambers, a mechanical stretch has been created on the alveolar-capillary barrier to mimic a human breathing lung. Figure is reproduced from Reference [12].

Gas exchange between air and blood takes place in the pulmonary system which has a complex structure. A bio-mimicking in vitro model of the lung is critical to model lung diseases (e.g., infectious diseases), drug development and toxicity tests. The most specific characteristic of the lung is its dynamic and periodic mechanical motion. In the pioneering work by Huh et al. [12], the first breathing lung-on-a-chip model has been introduced. In this biomimetic microdevice, the critical functional unit of the living lung (alveolar-capillary interface) has been constructed. To mimic the effect of breathing, this breathing lung-on-a-chip model used a lateral vacuum to apply cyclic stretching motion on a thin porous flexible PDMS membrane which acts as the interface between pulmonary microvascular ECs and alveolar epithelial cells (Figure 6). This model has been used to test immune responses to pulmonary infection and the response to nanoparticles. Interestingly, the periodic mechanical motions influenced the experimental data. This highlights the importance of physiologically relevant in vitro platforms to model human diseases and drug testing.

Douville et al. [82] developed an alveolar model. In this platform, alveolar epithelial cells were influenced by two different physiological conditions: the combination of solid mechanical stresses and surface-tension stresses, and exclusively cyclic stretch. The impact of fluid-filled alveolar cavities (e.g., in pneumonia) in alveolar epithelial cell death has been studied. The results demonstrated that cell detachment and cell death increased when cells experienced a combination of fluid and solid mechanical stresses.

Stucki et al. [83] presented a lung-on-a-chip model by the construction of an alveolar barrier in respiratory dynamics. A patient derived bronchial epithelial cell line demonstrated how mechanical stretch influenced epithelial barrier permeability. This model also showed that cell culture improved in the in vivo like dynamic model compared to a static one.

Recently, Jain et al. [84] developed a therapeutic model of microfluidic lung-on-a-chip for intravascular thrombosis in lung alveolus. Antagonist to protease-activated receptor-1 has been tested in this platform. This platform offers the potential to mimic pulmonary thrombosis pathophysiology towards antithrombotic drug development.

Benam et al. developed a “small airway-on-a-chip” model. This model has an air channel on top and a fluid flow channel on the bottom, between which a mucociliary bronchiolar epithelium layer has been placed. Asthma has been mimicked in vitro by exposing the epithelium to interleukin-13 (IL-13). This model was able to recapitulate in vivo like responses to therapeutics.

The global public health crisis linked to the 2019 novel Coronavirus (SARS-CoV-2 or COVID-19) pandemic and its socio-economic disaster [85] brings so much attention to the OOC community regarding the crucial importance of developing reliable models of lung-on-a-chip platforms for disease modeling and drug development purposes to tackle the continuous spreading of COVID-19 and other human respiratory viral infections. In the recent work by Si et al. [86] a human lung airway biochip was developed and lined by human lung airway epithelial cells and pulmonary microvascular endothelial cells. To investigate the potential of this lung-airway-on-a-chip to mimic lung physiology and pathophysiology, seven anti-viral therapeutics have been tested on this platform. This study demonstrated that OOC models are promising in vitro tools to emulate human lung responses to respiratory infections.

#### 5.1.2. Cardiovascular

Multiple design factors need to be considered to mimic the cardiovascular physiological microenvironment. Cardiac muscle tissue consists of organized cardiac muscle cells (or myocardium) and fibroblasts. Blood vessels also have complex structures including smooth muscle cells, endothelial cells and blood flow which result in fluid shear and vessel deformations. Microfluidic platforms are able to apply defined shear profiles into the cells cultured in the system. In addition, microfluidic platforms can take advantages of using syringe pumps and on-chip valves [87] or Braille display devices [88] to move fluid flow over the cultured cells to create more a physiologically relevant in vivo like blood vessel microenvironment. Cardiovascular diseases are one of the leading causes of death worldwide [89] Therefore, new drugs and therapies to treat or prevent cardiovascular diseases are urgently needed. Moreover, many drugs to treat other diseases have adverse side effects on the cardiovascular system [89,90] which highlights the importance of in vitro models of the cardiovascular system for drug testing.

**Figure 7:**
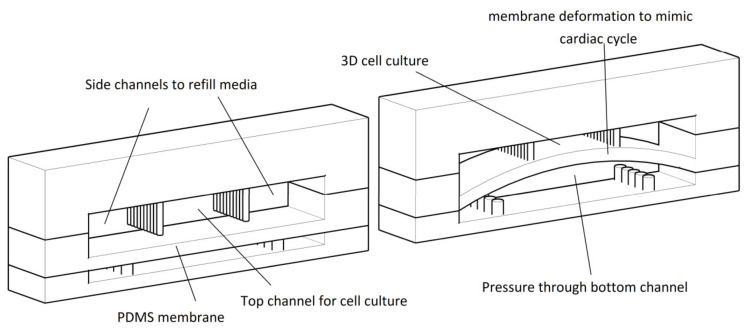
Schematic design of the heart-on-a-chip platform developed by Marsano et al. [92]. This includes two microchambers which are divided by a PDMS membrane. The top chamber is subdivided into a central channel to grow 3D cell culture, and two side channels to refill culture media. The cardiac muscle’s contraction and relaxation mimicked through deformation of PDMS membrane by applying pressure on the bottom channel. Figure is reproduced from Reference [92].

Au et al. [91] developed a polystyrene cell culture chip with microgrooves. Electrical stimulation applied and mutation and elongation of neonatal rat cardiomyocytes augmented to form gap junctions. Marsano et al. [92] fabricated a three-dimensional beating cardio tissue on a microfluidic chip (Figure 7). Mechanical stimuli which have been applied on the platform during the culture ended up with better cell maturation and augmented the electrical and mechanical coupling. Various concentrations of isoprenaline have been tested on this device which highlights the application of this heart-on-a-chip model in drug discovery and toxicology tests.

A mussel-inspired microfluidic chip was fabricated by Ahn et al. [93] to test cardiac contractility. Gelatins as an extracellular matrix as well as silver nanoparticles and titanium oxide have been used to fabrication of this three-dimension chip. In vitro contractile effects can be measured on this biomimetic analytical platform by cardiotoxicity of nanoparticles that affect calcium signal to sarcomere.

Lind et al. [94] used 3D printing to develop a cardiac tissue on a chip for drug testing applications. Xiao et al. [95] developed a microfabricated bioreactor to incorporate aspects of perfusion into the cardiac tissue model. Polytetrafluoroethylene (PTFE) tubing template, covered with neonatal rat cardiomyocytes (CMs) and human ESC derived CMs was used to create this perfusable 3D microtissue (which is called Biowire). This platform has been used for drug testing. Another cardiac model was developed by Ren et al. [96] to mimic hypoxia-induced myocardial injury. This microfluidic platform emulates the interface between cardiac tissue and blood vessels. By using the specific oxygen consumer blocking agent in the channels, hypoxic conditions were created. This resulted in morphological changes (e.g., cell shrinkage) and signs of apoptosis. Finally, Guenther et al. [97] fabricated an artery-on-a-chip platform to study the physiological response of the small blood vessel. In this microfluidic platform, small blood vessels have been placed within the channel, and then negative pressure to specific regions applied to clamp them in place. Various concentrations of biochemical solutions can be perfused in the channel. This platform mimics the complexity of the physiological structure of the blood vessels.

#### 5.1.3. Brain

The brain is one of the most sophisticated organs which comprises of a wide variety of cells. Human brain genetics and functions are significantly different than animals. Therefore, animal models only can give us a basic understanding of the brain functions and diseases [98]. Microfluidic brain-on-a-chip models have been developed to better mimic in vivo conditions including chemical, electrical and physical conditions of the human brain [6,99]. Here, we briefly review the current developments of microfluidic brain models.

Owing to the importance of axons in neurodegenerative disease, some researchers only focused on neuronal axons. A microfluidic platform has been developed by Taylor et al. for high-resolution axonal transport. Isolation and monitoring of axonal mitochondria and axonal growth have occurred in this platform [100]. A circular microfluidic chip presented by Park et al. [101] in which the soma compartment was located in the center with sealed microgrooves divided from the axonal compartment. A straight pathway for axonal growth resulted from these microgrooves. An image processing method to quantify the axonal growth has been developed by these researchers to address the issue of invasive sampling to characterize the growth of axons. Effects of various extracellular matrix (ECM) components (e.g., matrigel, collagen, laminin and etc.) have been assessed on axons growth and soma compartments separately. Based on which parts of the neurons are exposed to these biomolecules, they have different effect on axon growth.

A multilayer microfluidic platform fabricated has been developed by Park et al. [102] to mimic in vivo brain microenvironement for neurodegenerative diseases and high-throughput drug testing. The pluripotent human cells were grown on a chip to incorporate the blood-brain barrier. The cellular interactions between human fetal neural progenitor cells and the mature model have been assessed in this platform. By using an osmotic micropump, the effect of flow on neurodevelopment has been investigated. Kunze et al. [103] developed a microfluidic platform for neural cell culture to construct neural layers and 3D architecture. They described agarose–alginate mixtures which build multilayered scaffolds with layers of embedded primary cortical neurons apart from cell-free layers. To form concentration gradients, B27 supplementation has been delivered. This 3D scaffold based microdevice has the potential to be used for in vitro studies and drug testing.

A silicone elastomer brain-on-a-chip in vitro model was presented by Kilic et al. [104]. Neurospheroids were cultured on a microfluidic chip with flow control. Complex neural network and neural differentiation occurred on the platform by changing the flow. Toxic effects of amyloid-β in two different conditions (with and without flow) have been assessed in the system. The results showed that neurospheroids develop better in the dynamic conditions. Dauth et al. fabricated a multiregional brain-on-a-chip model [105]. Particular disease models can be developed on this platform. A decrease of firing activity and change in the amounts of astrocytes and particular neuronal cell types in comparison with separately cultured neurons was noticed in their research. The effects of phencyclidine (known as angel dust) which is a mind-altering drug have been investigated in this platform as well.

**Figure 8:**
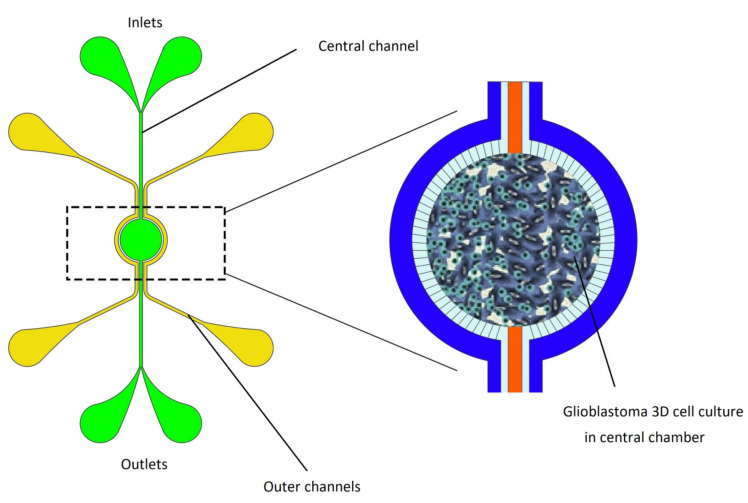
Brain tumor-on-a-chip model developed by Mimani et al. to access magnetic hyperthermia therapy on-a-chip. This chip was formed by a central and an external compartment separated by a porous interface. Magnetic nanoparticles through the central microfluidic channel introduced to central region of the chip which tumor cells have been cultivated in the 3D model. Then, through an alternating magnetic field, magnetic energy transforms to thermal energy and produce heat. Image reproduced from Reference [106].

Magnetic hyperthermia therapy attracted researchers’ attention in cancer therapy due to the generation of local heat to minimize damage to healthy cells nearby and optimize the treatment. Recently, a microfluidic based brain tumor-on-a-chip model was developed by Mimani et al. [106] to access the therapeutic effect of magnetic hyperthermia on the chip. A 3D cell culture of glioblastoma has been cultivated in the central zone of the microfluidic channel. Cell viability after exposure to magnetic hyperthermia therapy has been studied on the device. Results of fluorescence imaging have demonstrated that cell viability decreased by 100% after 30 min of exposure to magnetic hyperthermia therapy. However, tumor vasculature is absent in this model; the results of this study showed that this brain tumor OOC model has great potential to emulate the characteristics of glioblastoma brain tumor in vitro (Figure 8).

#### 5.1.4. Liver

The liver with its crucial functions such as metabolization, detoxification of blood from various metabolites and production of biomolecules for digestion, is one of the most vital organs. Since toxicity of the liver is one of the main causes of drug failure, an in vitro model of the liver that can mimic the in vivo like microarchitecture of the liver is crucial in drug development processes. Hepatic lobule (which is comprised of sinusoids and blood vessels lined with endothelial cells) is the functional unit of the liver [107]. Since the perfusion of fluid is the main characteristic of the liver, microfluidic cell culture platforms must provide perfusion conditions that better emulate liver function in vitro when compared to conventional cell culture models [108].

Powers et al. [109] developed a liver-on-chip model which had a silicon sheet scaffold with an array of channels which were separated by a microporous filter to provide upper and lower chambers for perfusion of the culture medium. They observed morphogenesis of 3D tissue structures under continuous flow perfusion in this platform. Lee et al. [110] presented a microfluidic liver-on-a-chip platform to assess hepatocytes interactions and hepatic stellate cells in 3D culture model. This platform was able to provide the continuous perfusion of culture medium to the cells through an osmotic pump without the need for an external power source. They developed monoculture and co-culture of hepatocytes and hepatic stellate cells and investigated cellular interactions with or without flow.

Lee et al. [111] introduced an artificial microfluidic liver sinusoid on a chip by packing a high density of hepatocytes into a microchannel. This platform recapitulates the transport phenomena in sinusoid in which sinusoidal endothelial cells facilitate blood and plasma transport to the hepatic cords. Transport properties of the sinusoid (e.g., continuous nutrient exchange and cell–cell interactions) were maintained in this platform to give the possibility to primary rat and human hepatocytes to stay viable for a period over 7 days. By using a liver toxicant, hepatotoxicity has been assessed in this liver sinusoid on chip platform.

Bavli et al. [112] developed an integrated microfluidic liver-on-a-chip platform to monitor metabolic activities (e.g., glucose uptake, lactate production and oxygen uptake) in vitro. Khetani et al. [113] introduced a model in which hepatocytes and 3T3 fibroblasts were cultured on a micropatterned collagen in 24-well plates. Cho et al. [114] presented microfabrication and micropatterning techniques to create layered hepatocytes on micropatterned fibroblast feeder layers. This platform has been used to assess cellular interactions in the co-culture model of hepatocytes and 3T3-J2 fibroblasts.

In the liver-on-a-chip model presented by Delalat et al. [115], a 3D cellular microstructure was developed to mimic the hepatic sinusoid for screening the drug cytotoxicity. Furthermore, some other liver-on-a-chip platforms have been developed for in vitro modeling of liver injury and disease [116,117].

Recently, Kamei et al. [118] introduced a simple 3D liver-on-a-chip model with mature hepatocyte-like cells which have been differentiated from human pluripotent stem cells (hPSCs). In this study, a microfluidic cell culture platform was used for the hepatocyte-like cells maturation. This platform can be served in drug testing and chemical-safety assays.

#### 5.1.5. Kidney

The Kidney is one of the most sophisticated organs to mimic since it is comprised of several tissues. The kidney is known as a vital organ for its crucial functions including filtration of blood, toxin removal and maintenance of electrolyte balance. One of the most reported adverse effects during the drug development process is the toxicity of the kidney. The first model of toxicity was a study on a kidney-on-a-chip microdevice reported by Jang et al. [119]. In this model, primary kidney proximal tubular epithelial cells have been cultured on a microfluidic device under perfusion. Fluidic flow emulates the key characteristic of the human kidney proximal tubule. Reabsorption of glucose, transport of albumin and alkaline phosphatase activity have been studied in this microfluidic kidney-on-a-chip model.

According to the importance of glomerulus which is critical in blood filtration, Musah et al. [120] developed a kidney-glomerulus-on-a-chip model. In this platform, human induced pluripotent stem cells differentiated into podocytes which are the cells that regulate selective permeability in glomerulus. Glomerular basement-membrane collagen to provide tissue–tissue interactions with glomerular endothelial cells have been generated in vitro. Adriamycin-induced albuminuria and podocyte injury have been emulated via this glomerulus-on-a-chip model.

A glomerulus-on-a-chip microfluidic platform developed by Zhou et al. [121] emulated the vascular and epithelial interface between podocytes and endothelial cells in the kidney glomerulus. To mimic the glomerular microenvironment in vitro, perfusion of fluid flow and mechanical forces have been applied in this microdevice. This study demonstrated the importance of shear stress and hydrodynamic pressure in cellular cytoskeletal rearrangement and cellular damage to emulate kidney disease such as hypertensive nephropathy.

Recently, a virus-induced kidney disease model was fabricated by Wang et al. [122] by developing a three-layer microfluidic platform. This distal tubule-on-a-chip model was presented to investigate the pathogenesis of virus induced renal dysfunction in regulation of electrolyte. 

A review published by Wilmer et al. [123] focuses on development of kidney-on-a-chip technology to mimic the structural and functional properties of the human kidney for prediction of drug-induced kidney injury.

#### 5.1.6. Gut

Human in vitro models of the intestine are very important in pharmacokinetics studies. Conventional in vitro models fail to recapitulate physiological microenvironment (e.g., cyclic prestaltic motion) of the human gut. Therefore, microfluidic platforms provide a powerful in vitro model of intestine to mimic in vivo like microenvironment for drug testing.

A microfluidic device consisting of two lumens which have been separated by a porous membrane was developed by Kimura et al. [124]. A stirring pump was integrated to the platform to control the fluid flow in the system and to create a dynamic environment for the cells. Caco-2 cells have been cultured in the system for over 30 days and Rhodamine 123 was added into the system to investigate the cells permeability.

In the microfluidic gut-on-a-chip model developed by Kim et al. [125] provides in vivo like peristaltic movements and fluid dynamics were applied on the system to induce intestinal epithelial cells to undergo multiple intestinal cell types differentiation. The 3D structures and complex physiological functions of the human intestine can be recapitulated in this platform. An intestinal inflammation model was developed on a gut-on-a-chip platform presented by the same group. The platform can be used for the pathophysiological study of human intestinal inflammation produced by overgrowth of bacteria [126].

Recently, a gut-on-a-chip model was developed by Shin et al. [127] which provides mechanical movements and perfusion of fluid flow in the system to investigate the role of physical stimulus on intestinal morphogenesis. Human intestinal Caco-2 and primary intestinal epithelial cells have successfully been cultured in this gut-on-a-chip model. Other cell types such as mesenchymal cells can be incorporated to this system to investigate how they can contribute to intestinal morphogenesis. The 3D morphology which was observed in this model, matches perfectly with the related computational simulations.

#### 5.1.7. Skin

The skin is considered as the largest organ with several specific functions including regulation of body temperature, prevention of dehydration and acting as the first protection shield to protect other organs against environmental stressors (biological, physical and chemical). For toxicology testing of new compounds, physiologically relevant skin models are of crucial importance for pharmaceutical, chemical and cosmetic industries to identify potential hazards on the skin [128]. In this section, we highlight the current developments on skin-on-a-chip models. One of the key features to develop biomimetic skin models is to simulate vasculature and blood circulation in vitro. Thus, the combination of tissue engineering with microfluidic technology by providing the possibility of media perfusion is expected to reconstitute more relevant skin models and provide valuable evaluation of drug tests [129].

Wagner et al. [130] developed a microfluidic platform integrated with a peristaltic micropump for co-cultures of human artificial liver microtissues and skin biopsies. In long-term (14 days) exposure to fluid flow, crosstalk in the co-culture has been observed. In this platform, tissue sensitivity by exposure to troglitazone, a pharmaceutical substance, has been investigated. In vitro skin models which consist of dermal and epidermal layers are more physiologically relevant when compared to single epidermal layer models. A skin-on-a-chip platform designed by Abaci et al. [131] had a specific capability of recirculating the media without the need for pumps or external tubing at favorable flow rates. Physiological residence time of blood in the skin tissue has been established in this platform to provide relevant concentration of drugs in the blood. This platform is used for toxicity drug testing.

The incorporation of biosensors with biochips provides in situ and real-time monitoring of skin tissue responses to the test item. A sophisticated skin-on-a-chip platform integrated with a sensor was developed by Alexander et al. [132] for monitoring the transepithelial electrical resistance of recreated human epidermis. Metabolic parameters and change of skin tissue over time have been monitored in this platform. Recently, Kwak et al. [133] fabricated a skin-on-a-chip microfluidic platform in which epidermal and dermal layers were co-cultured with human umbilical vascular endothelial cells. Immune responses such as an increase in secretion of cytokines and migration of neutrophils into the demal layer after exposure to doxorubicin and UV irradiation have been observed.

A three-layer PDMS microfluidic skin-on-a-chip device with two porous membranes was fabricated by Wufuer et al. [134] In this model, a co-culture of human skin cells (epidermal, dermal and endothelial layers) has been developed. Separate microfluidic channels in this system provide the possibility of perfusion of various kinds of media with different flow rates. By perfusion of tumor necrosis factor alpha into the channels, skin inflammation and edema have been mimicked in this system. Drug toxicity testing by studding dexamethasone’s effect on reducing inflammation and edema has been performed in this model. Although this dynamic and multilayer co-culture platform lacks the 3D microenvironment of the skin.

Lee et al. [135] developed a skin-on-a-chip model based on 3D co-culture. In their design, dermal primary fibroblasts have been embedded in hydrogel to provide a 3D dermal layer. Then, on top of the collagen-fibroblast layer, primary keratinocytes have been cultured to represent the epidermal layer. Endothelial cell-coated microfluidic channels have maintained the growth and differentiation of skin cells in this biochip. Cell culture experiments combined with mathematical modeling have demonstrated that perfusion through a microfluidic network maintains the long-term culture of skin cells for up to two weeks. A skin-on-a-chip platform has been presented by Jusoh et al. [136] to mimic the effect of skin irritants on angiogenesis. In this model, irritated keratinocytes biochemically stimulate vascular endothelial growth factors. Autocrine and paracrine interactions between dermal fibroblasts and keratinocytes increase angiogenic sprouting in this model. The effect of the sodium lauryl sulphate (a well-known chemical irritant) and steartrimonium chloride (which is known as a non-irritant compound) has been studied in this platform.

More recently, a simplified gelatin-based skin-on-a-chip model has been developed by Jahanshahi et al. [128] for studying wound infection, skin’s pro-inflammatory response and drug screening. In this platform, keratinocytes have been cultured on the microchannels which have been embedded in a gelatin matrix. After long term (6 weeks) culture, a multilayer structure of epidermis layer was formed. However this model lacked the presence of other cell types (e.g., fibroblasts). It is still a functional model to study the skin’s pro-inflammatory responses to bacterial infection and drug testing.

As briefly reviewed here, in the past few years, various skin-on-a-chip models have been fabricated. However, to develop more reliable skin models to mimic the complexity of 3D architecture of human skin, the presence of vascular network and immune cells is crucial for toxicology tests and study skin diseases.

### 5.2. Body-on-a-Chip

Multi-OOC platforms or body-on-a-chip platforms refer to in vitro models which emulate interactions between two or multiple human organs within a microfluidic system. These complex microfluidic platforms can be used to emulate interactions among divers organs for drug discovery, toxicity tests etc. [137]. There are some biological challenges such as creating a suitable media for all cell types, appropriate scaling of organs, immune responses in the system etc. that need to be considered in multi-OOC platforms. Moreover, technical challenges including avoiding bubble formation in these complex systems, maintaining long term sterility, optimizing the physiological parameters for different organs etc. need to be addressed in these sophisticated microdevices. References [137,138] highlight the critical design parameters for biomimetic multi-OOC platforms to develop physiologically based pharmacokinetics (PBPK) and pharmacodynamics (PD) models which have the potential to be used in the pharmaceutical industry.

Since liver and kidney are the two crucial organs in metabolism and excretion of drugs, a liver–kidney co-culture model [139] was developed on a microfluidic platform to investigate metabolism changes under drug components. HepG2/C3A cells co-cultured with kidney cells for toxicity test of a variety of molecules. This platform can be used in pharmaceutical and environmental toxicology testing. A liver-kidney model has been presented by Choucha-Snouber et al. to investigate the toxicology of an anticancer agent (Ifosfamide). By comparing the results of the liver–kidney co-culture with the kidney monoculture biochip, this study highlights the importance of multi-organ microfluidic in vitro models in toxicity tests and drug testing.

An integrated liver–heart–vascular microdevice developed by Vunjak-Novakovic et al. [140] incorporated liver, cardiac and blood vessel cells in a microfluidic platform which potentially can be used in toxicity tests of cardiovascular drug components.

In another interesting study which was presented by Maschmeyer at al. [141] Troglitazone, an antihyperglycemic and anti-inflammatory drug which was withdrawn from the market because of its toxicity effects on liver, has been tested on microfluidic platforms. The liver toxicity was observed in response to Troglitazone on two separate microfluidic co-culture platforms (liver-intestine and liver-skin) which highlights the efficacy of in vitro multi-OOC models in drug testing.

A microfluidic liver-intestine model introduced by Bricks et al. [142] demonstrates the critical role of intestine in oral medications absorption and excretion. This model emulates the interactions between HepG2 C3A cell lines and intestinal Caco-2 TC7 cell lines to assess intestinal absorption and liver metabolism of drug components. They studied the interactions between liver an intestine cells in a conventional co-culture model and a microfluidic co-culture biochip. The results demonstrated that the functionality of the liver in microfluidic biochip increased when compared with conventional models. Moreover, drug metabolism was elevated in the biochip co-culture platform when compared to co-culture in Petri dishes and with monoculture on a microfluidic platform.

A four-OOC model was presented by Maschemeyer et al. [143] to study absorption, distribution, metabolism and excretion in co-culture model of intestine, liver, skin and kidney. Other multi-OOC platforms have been reported by several research groups word wide including liver-tumor-bone marrow [144], liver-lung-adipose tissue-other tissue [145], liver-lung-kidney-adipose tissue [146], liver-vascular -adipose tissue [147,148].

### 5.3. Organ-on-a-Chip Market

While various challenges [6,33,149] remain to be addressed before adoption of OOC technology by clinicians and pharmaceutical industry, a number of start-ups have been raised in the past few years to occupy the high-potential market by their own innovative technology. Some start-ups are more specialized in developing specific OOC, while others propose to develop an independent biochip with the possibility of developing different organs. Some others have the intention to fabricate a single organ device and some others prefer multi-organ platforms to assess interactions between organs. Each player in the field proposes its own unique technology, which has specific applications, drawbacks and strengths. However, still there is a significant gap between market needs and available technologies and it seems new players need to show noticeable progress to demonstrate the predictive validity and reproducibility of OOC platforms for disease modeling and drug discovery. The adoption of OOC technology by industry will start with contract research organizations (CROs), which support biotechnology and pharmaceutical industries in R&D services [150]. Therefore, OOC companies require abundant quantitative and qualitative research to prove that these OOC devices are true mimics of human organ functions and accelerate their adoption by CROs and consequently by industry and health care system [33]. Here, we have briefly summarized a number of pioneers in commercialized OOC devices (Table 3). Interested readers refer to Reference [33] for extensive details and discussion in this regard.

## 6. Discussion and Future Perspectives

This state of art summarizes current progress regarding BioMEMS, LOC for POC applications, OOC platforms and the history of developing these technologies specially for new players in the field. Indeed, this review is dedicated to background and current advancements in LOC and OOC technologies and their applications in life. Since the main intention of developing biomedical devices is to bring the technology from academia to the market for improving human health, a list of pioneers in commercialization of LOC and OOC devices has been summed up for interested readers. Here, having a look at developed LOC devices for POC applications and OOC platforms by considering their specifications, limitations and strengths presented above and summed up in Table 4, we highlight possible future perspective for OOC technologies.

It has therefore been shown that materials, microengineered technologies, functionality, reproducibility, being user friendly and automation through integrated systems are the main parameters involved during development of each one of these inventions. Although many features of these technologies have been described in the literature and summarized in the present work, numerous others have to be investigated further.

MEMS based devices have a wide variety of applications from the electronic industry [2] to environmental [151] and biomedical fields [1]. Development of commercialized BioMEMS including LOC for POC devices and microfluidic biochips continue to increase consistently. Various exciting directions are expected for the future of LOC highly integrated devices for diagnostic and therapeutic are some of the interesting applications of LOC devices. For instance, rapid detection of viral infections [152,153] and early detection of cancer biomarkers [154,155] directly from body fluids are some of the highly attractive subjects in this area.

According to the literature, some of the LOC devices have been already commercialized years ago (e.g., home pregnancy tests, real-time glucose monitoring, etc.). Miniaturized portable LOC devices for POC applications are ideally suited for testing in developing countries where expensive and complex laboratories do not exist. These automated, accurate and cost effective diagnostic devices do not need highly trained health care experts and can be used on-site by patients. In most of the LOC devices which are categorized as a subset of BioMEMS, electrical and/or mechanical parts are integrated to the system [20,63]. Therefore, in these platforms, normally whole patient samples (e.g., blood, saliva, urine and etc.) are introduced into a single chip and all the steps of the laboratory process including separation, filtration, analysis and read out of the results take place on the small single chip. The result of the assay usually would be available visually (e.g., yes or no in pregnancy tests or digital numbers of the blood glucose level in glucometers) and hence it does not need inferences by the experts. The global health crisis due to SARS-CoV-2 (COVID-19) [156] pandemic highlights the critical importance of LOC for POC testing for early diagnosis of suspected cases and treatment monitoring of infected people to prevent further spread of the infection. Real-time reverse transcriptase polymerase chain reaction (rRT-PCR) is the current diagnostic approach for COVID-19 [157]. However, this is a time-consuming and costly method which requires developed facilities and highly skilled staff and is not robust in the early stages detection and can show false negatives for up to two weeks. Hence, rapid, sensitive and automated LOC devices for POC applications are urgently needed to detect COVID-19 from patient samples (e.g., saliva, plasma and etc.) [85,158].

Since cell culture is a critical aspect in biomedical science for understanding human physiology and pathophysiology, toxicology tests, drug screening etc., microfluidic-based cell culture platforms with the aim of mimicking the in vivo like microenvironment for cells have strongly attracted researchers’ interest in recent years worldwide. However, most of the microfluidic-based cell culture devices, which have been developed in academia, are not integrated with electrical or mechanical parts, but in terms of microfabrication technologies and principals, these platforms, which are well known as OOC platforms, have been categorized as a subset of BioMEMS systems. These platforms take advantage of microfluidic systems and microfabrication techniques to provide cell culture and tissue culture platforms which recapitulate tissue–tissue interactions, biochemical and biomechanical microenvironments of living organs. OOC systems use cells, spheroids, organoids and tissue biopsies to mimic tissue or organ level functionality in vitro. Hence, unlike LOC platforms, these samples are required to be prepared by trained experts and the results of the analysis need to be concluded by scientists in the field. Most of the microfluidic OOC platforms are still in the proof-of-concept stage because of some major challenges including low throughput (e.g., due to single channel designs), short lifespan of the biochip (e.g., due to channel blocking or clogging), complex sample preparation and limitations linked with material for fabrication (e.g., absorption of small molecules by PDMS). Making balance between academic research and commercialization is worthwhile to expand microfluidic based OOC platforms into clinically useful in vitro tools. Achieving organ physiological function in vitro is not an easy process. Another key point to develop commercialized OOC devices is the design of the device. An appropriate design for microfluidic OOC platform needs to be simple enough for the ease of manufacturing and operation for the end users, while having an adequate complexity to mimic the sophisticated structure and physiological function of an organ or a tissue to be adopted by the market. Although, based on the works presented above, it can be established that the state of knowledge is currently insufficient to design efficient and realistic mimics. Automation and integration of OOC devices is another important aspect to be considered for transferring OOC technology from discovery to broad availability and adoption by industry for drug toxicity and efficacy tests. Indeed, commercialized OOC devices need to be multiple-organ-on-a-chip platforms which are integrated with various sensors (e.g., physical and electrochemical sensors) for real-time and continuous measurement of micro-environmental parameters (e.g., temperature, pH, oxygen level, soluble biomarkers, drug concentration and etc.) during a long period of an assay (7 days or more). Integration of a miniaturized microscope with such a platform, by providing in situ monitoring of morphological changes, offers a versatile tool for clinicians, biotechnology and pharmaceutical industries [149]. However, to achieve this goal, effective collaboration between clinicians, scientists and engineers is required.

Advances in OOC technology have increased the hope for personalized medicine and POC testing. Since disease modeling and drug screening are the two main goals of OOC platforms, by using patient-derived samples (cells, tissue biopsies etc.) on these platforms, we can develop individual patient-on-a-chip models for POC application. Indeed, personalized patient-derived OOC platforms have the potential to accelerate time-consuming and expensive drug discovery processes. Hence, fast detection of the best approach for treatment brings personalized health care for individuals with genetic differences [6]. For instance, targeted drug delivery [159] and combination therapy [160,161] have gained great interest in cancer therapy to develop novel treatments. Direct and localized delivery of drug molecules to specific tissues or organs in the body enhances desired therapeutic concentration in the targeted tissue, while minimizing systematic drug side effects [159]. In combination therapy, clinicians combine two or more therapeutic agents or approaches to discover a patient’s optimal treatment [160]. OOC platforms have the potential to play a critical role in the future to advance combinational therapy and targeted drug delivery assays specially for diseases like cancer, which time is crucial and there is no place for trial and error on the patient.

Finding new therapeutics for rare diseases [162,163], which affect a small population, is another expected application for OOC devices in the future. Small market size and high expenses to develop new treatments for rare disease are the main reasons that most pharmaceutical companies are not interested in investing to develop treatments for rare diseases. However, in recent years, advances in biomedical technologies have increased the attention of governments and the pharmaceutical industry for drug discovery for rare diseases. Personalized OOC devices using patient’s samples allow us to conduct trials on a biochip to tackle the challenges of therapeutic discovery for rare diseases.

As mentioned above, automation and integration of OOC devices with different sensors and microscopes by in situ and real-time data collection decrease user interference and facilitate the device’s operation. Therefore, integrated patient multi-organ-on-a-chip platforms have the potential to be used by pharmaceutical companies and clinicians for POC applications. As reviewed above, these patient-on-a-chip platforms are required to recapitulate the complexity of living organs and organ–organ interactions while keeping the device simple to promote the operation by users.

## 7. Conclusions

Along with advancements in microtechnologies and the development of LOC devices in life science, particularly for POC applications, microfluidics have merged seamlessly with tissue engineering to develop OOC platforms which use building blocks of human organs on biochips to mimic organ physiology and to recapitulate organ functionality in vitro. However, there are still many challenges to be addressed before OOC technology finds its own place among other preclinical methods used by CROs, the pharmaceutical industry and clinicians. This requires effective and close collaboration between scientists, clinicians and engineers to bring this technology to the POC.

We believe that future OOC devices for POC applications need to be multi organ platforms which are personalized and use patient-derived cells or tissue biopsies to recapitulate the complexity of each patient on a biochip while keeping this complexity clinically relevant to facilitate their adoption by clinicians and pharmaceutical pipeline to accelerate patients’ treatment.

## Figures and Tables

**Table 1:** List of selected microfluidic companies working to manufacture integrated point-of-care (POC) diagnostic devices. Adapted from Reference [41].

Company Name	Materials and Manufacturing	Analytes	Applications	Sample Types	Signal Detection	Highlights of Technology Suited for POC
Abaxis	Plastic disc	Small molecules, proteins	Blood chemistries (e.g., metaboliteselectrolytes)	Whole blood	Absorbance	Compact analyzer, injection-molded plastic discs, no pre-processing of sample
Advanced Liquid Logic	Glass, insulated electrodes	Small molecules, proteins, nucleic acids	HIV/AIDS, Iysosomal storage disease	Whole blood	Fluorescence, chemiluminescence	Sample pre-processing compact, benchtop analyzer, manipulation of nano-and micro-droplets
Alere(formerly inverness Medical)	Plastic and elastically deformable materials	Proteins, cells	HIV/AIDS, clotting time	Whole blood	Fluorescence	Disposable cartridge, portable analyzer, automated image-based immune hematology test
Biosite (Alere)	Strip with textured microstructures	Small molecules, proteins	Cardiovascular disease, drugs of abuse, waterborne parasites	Whole blood, plasma	Fluorescence	Portable reader, disposable capillary-driven microfluidic test strips
Cepheid	Disposable plastic cartridge	Nucleic acids	Respiratory infections (bacterial and viral), cancer	Whole blood, sputum	Fluorescence (with molecular beacons)	Disposable cards with benchtop analyzer, on-card sample processing (sputum)
Daktari Diagnostics	Plastic cartridge	Cells	HIV/AIDS	Whole blood	Electrochemical (impedance spectroscopy)	Handheld instrument, label-free electrochemical sensing of captured cell lysate
Diagnostic For All	Paper	Small molecules, Proteins	Liver damage from HIV/AIDS medication	Whole blood, urine	Colorimetric	Instrument-free tests based on paper, capillary-driven microfluidics, colorimetric readout
Epocal (Alere)	Film, epoxy laminates	Small molecules	Blood chemistries	Whole blood	Electrochemical, chemiluminescence	Self-contained cards, patterned electrodes for sensing, wireless data transmission
Focus Dx (Quest)	Plastic (polypropylene)	Nucleic acids	Flu, intestinal pathogens	Nasal and pharyngeal swabs	Fluorescence	Portable detector, discs with on-board extraction
HandyLab (BD)	Disposable cartridges	Nucleic acids	Bacterial infections and drug susceptibility testing	Vaginal, rectal, nasal swabs	Fluorescence (with molecular beacons)	Disposable cards with integrated heating, detection, sample processing in a portable instrument
i-STAT Corp (Abbott)	Plastic cartridge with silicon microchip	Small molecules	Blood chemistries, coagulation, cardiac markers	Whole blood, urine	Electrochemical (potentiometry, amperometry, conductivity)	Portable analyzer, capillary-driven microfluidics, thin-film electrodes for detection
Micronics (Sony)	Plastic, laminates, paper	Proteins, nucleic acids	Malaria, shiga toxin-producing E-coli, ABO blood typing	Whole blood, stool	Absorbance, colorimetry	Disposable cartridges composed of thin-film laminates and injection-molding
Philips	Plastic cartridge	Nucleic acids, small molecules, proteins	Cardiac damage, drugs of abuse, hormones	Whole blood, saliva	Optical (frustrated total internal reflectance)	Handheld reader with self-concentration of magnetic nanoparticles for rapid analysis
TearLab	Polycarbonate	Small molecules	Dry eye disease (tear osmolarity), ocular allergy (IgE antibodies)	Tear	Electrochemical	Portable osmolarity reader with disposable cards; capillary-driven flow, gold electrodes for detection, results in 5 s

**Table 2:** A comparison between the advantages and disadvantages of macroscopic cell culture models and microfluidic cell culture platforms.

Cell Culture Methods	Advantages	Disadvantages
**2D cell culture**	▪ Well established methodology ▪ Easy to work ▪ Cost effective	▪ Lack of 3D environment for cell-cell and cell-ECM interactions ▪ Uniform concentration of nutrients and drugs ▪ Fail to produce dynamic microenvironment ▪ Not enough biological relevant model
**3D cell culture models**	▪ Provide 3D environment for cell-cell and cell-ECM interactions ▪ Present of soluble gradient (nutrients and drugs) ▪ Provide more physiologically relevant model for drug testing	▪ Extra expenses ▪ Fail to produce dynamic microenvironment ▪ Lack of fluid flow perfusion ▪ Expensive ▪ Challenges in microscopy and measurement
**Microfluidic cell culture platforms**	▪ Precise control over cellular microenvironment ▪ Diffusion gradient of nutrients and drugs ▪ Ability to provide fluid flow ▪ Produce in-vivo like and dynamic environment ▪ Cost-effective(in term of less reagents consumption) ▪ Less sample needed (advantage for personalized medicine) ▪ Provide fast test results ▪ Possibility of merging with mechanical stimuluses and sensors ▪ Real time and on-chip analysis ▪ Custom made device	▪ Non-standard protocols ▪ Expenses and difficulties associated with fabrication ▪ Bobble formation in channels ▪ Channel clogging by cells ▪ Complex design and manufacturing process and challenges associate with operational control

**Table 3:** List of some of the OOC start-ups and highlight of their technology.

Company’s Name	Summary of Specialties	Applications	Cell Source	Highlight of the Technology	Year
**AlveoliX**	OOC, Lung-on-a-chip	Drug discovery, disease modeling	Human cell lines	In vitro models inspired by nature, reproduce lung breathing motion, elastic and ultrathin membrane	2015
**AxoSim**	OOC, nerve-on-a-chip	Preclinical testing, 3D cell culture, neurotoxicity tests, neurodegenerative diseases	Primary cultures, Organoids	Biomimetic human tissues, combination of neurons, astrocytes, and oligodendrocytes.	2014
**BEOnChip**	OOC	Disease modeling, in vitro tests, drug screening	Human cells	Long-term 2D or 3D culture under flow condition, 2D-3D co-culture, simulation of physiological environments involving flow and shear stress	2016
**BiomimX**	OOC, heart-on-a-chip, Cartilage-on-a-chip	Drug screening, drug cardiotoxicity, anti-arrhythmic drug efficiency, discovery anti- osteoarthritic drugs	cardiomyocytes derived from human iPSCs, human cells	3D co-culture, mechanical stimulations, human cardiac tissue, human osteoarthritic cartilage, customized OOC	2017
**BI/OND**	OOC, BI/OND’s microfluidic plate	In vitro tests, Drug discovery	Human cells, Organoids, patient derived cells or tissues	Dynamic cell culture environment by providing mechanical stimulation and continuous fluid flow, two compartments connected by a porous membrane BI/OND’s plate to run up to six cultures in parallel, 3D and 2D models	2017
**CN Bio Innovations**	OOC, ), liver-on-a-chip, Body-on-a-chip (7-OOC )	Human physiology modelling, liver diseases modelling, Preclinical drug discovery, toxicity tests, drug metabolism	Primary human cells, Tissue or Organ Slices, IPSCs, Immortalised cell lines	Multi organ studies, portable and compact device, programmable flow rate, open well plates	2009
**Emulate, Inc.**	OOC, Lung, Bone marrow, kidney, brain, blood vessels and intestine-on-a-chip	Personalized medicine, disease modelling, drug screening, study human physiology	-	Organ-Chips personalized with individual patients’ stem cells, stretchable biochip, flexible and dynamic environment by fluid flow and mechanical stretch	2014
**Hesperos**	OOC, multi-organ-on-a-chip ( heart, liver, lung, brain, skin, muscle, kidney, pancreas, bone marrow)	In vitro tests, drug discovery, toxicity tests, pharmacokinetic/ pharmacodynamic modeling	human stem cells	Pumpless platform, recreate muscle and tissue function, neural and inter-organ communication, customized human-on-a-chip platform, possibility to add immune cells in multi-organ-platform	2015
**MIMETAS**	OOC	Disease modelling, drug testing, toxicity tests, personalized medicine	Human cells, patient derived cells or tissues	OrganoPlates (a microfluidic 3D cell culture plate), 3D co-culture, biomimetic, compatible, easy to use	2013
**Nortis**	OOC, kidney, brain, heart, liver, immune system and blood vessels-on-a-chip	Disease modeling, cancer study, drug testing, study Alzheimer’s disease and ageing, toxicity tests	Human derived tissue models	Perfusion system, standard cell culture incubator,	2012
**SynVivo, Inc**	OOC, blood-brain-barrier-on-a-chip	Drug discovery, toxicity test, targeted drug delivery, cancer researches	Human cells	Mimic microvascular environment, dynamic environment, real-time visualization, controlled condition, 3D co-culture model	2014
**TARA Biosystems**	OOC, heart-on-a-chip	Cardiac Toxicology, Precision Cardiology, Heart Failure Drug Discovery, Drug development, study healthy and disease models	iPSCs derived cardiomyocytes	Cardiac tissue models, patient derived disease models	2014
**TissUse**	OOC, body-on-a-chip	Toxicity tests, disease modeling, personalized medicine, drug development, application in pharmaceutical and cosmetic researches	Cell lines, human primary cells, biopsies	Multi-organ platforms, rapid prototyping, compatible with tissue imaging, application of physiological sheer stress, long-term performance	2010

**Table 4:** A summary of specifications, limitations and strength of current LOC and OOC.

Technology	LOC	OOC
**Applications in life science**	Recapitulate one or several laboratory functions on a single chip; POC diagnostic devices (e.g., hormone detection, viral infection detection and etc.)	Study human physiology; Disease modeling; Drug screening and development; Toxicity tests; Personalized medicine
**Advantages**	Integration with miniaturized sensors, valves and pumps; Use microfabtication techniques (e.g., photolithography, etching, 3D printing and etc.); Possibility to collect data with non-trained individuals; Small size and being portable; Low volume of sample and reagents consumption; Quick sample analysis; User friendly; Automation opportunities	Ability to be integrated with miniaturized sensors and actuators; Advantages linked with new microfabtication techniques (e.g., softlithography, 3D bioprinting and etc.); Small volume of sample and reagents consumption; Real-time and on chip analysis; Precise control over microenvironment
**Drawbacks**	Problems linked with traditional microfabrication techniques (costly, time consuming, need for highly trained experts for fabrication and etc.); Complex configuration with multiple pumps, valves and sensors; Problems associated with microliter scale due to surface dependent effects (e.g., capillary forces and surface roughness)	Need well-trained experts to collect and interpret data; Current problems with fabrication materials (e.g., absorption of small molecules by PDMS) Current platforms mostly are not automated; Problem linked with cell clogging and bubbles formation in microchannels; Non-defined protocols; cell damages due to shear stress
**Size**	Few square millimeters to few square centimeters	Few square centimeters
**Materials**	Silicon, glass, metal, ceramic, polymers, thermoplastics, paper and etc.	Biocompatible and cytocompatible materials such as polymers (e.g., PDMS); glass; biological materials (e.g., proteins, cells etc.)
**Biological samples**	Body fluids (e.g., Saliva, blood, urine)	Cells, spheroids, organoids, tissue biopsies

## List of Acronyms

BioMEMS	Biomedical Micro-Electro-Mechanical Systems
CE	Capillary Electrophoresis
CMs	Cardiomyocytes
CROs	Contract research organizations
ECM	Extracellular Matrix
GC	Gas Cromatography
GPC	Gas-phase chromatography
hCG	human Chorionic Gonadortropin
HPLC	High-Pressure Liquid Chromatography
hPSCs	human Pluripotent Stem Cells
iPSCs	Induced-pluripotent stem cells
IC	Integrated Circuits
IL	Interleukin
LOC	Lab-on-a-chip
MEMS	Micro-Electro-Mechanical Systems
OOC	Organ-on-a-chip
PBPK	Pharmacokinetics
PD	Pharmacodynamics
PDMS	Polydimethylsiloxane
POC	Point-of-Care
PTFE	Polytetrafluoroethylene
R&D	Research and development
UV	Ultraviolet
μTAS	Micro-Total Analysis Systems
